# Brain Metastases Research 1990–2010: Pattern of Citation and Systematic Review of Highly Cited Articles

**DOI:** 10.1100/2012/721598

**Published:** 2012-09-17

**Authors:** Carsten Nieder, Anca L. Grosu, Minesh P. Mehta

**Affiliations:** ^1^Department of Oncology and Palliative Medicine, Nordland Hospital, 8092 Bodø, Norway; ^2^Institute of Clinical Medicine, Faculty of Health Sciences, University of Tromsø, 9038 Tromsø, Norway; ^3^Department of Radiation Oncology, University Hospital Freiburg, 79106 Freiburg, Germany; ^4^Department of Radiation Oncology, Northwestern University, Chicago, IL 60611, USA

## Abstract

*Background*. High and continuously increasing research activity related to different aspects of prevention, prediction, diagnosis and treatment of brain metastases has been performed between 1990 and 2010. One of the major databases contains 2695 scientific articles that were published during this time period. Different measures of impact, visibility, and quality of published research are available, each with its own pros and cons. For this overview, article citation rate was chosen. *Results*. Among the 10 most cited articles, 7 reported on randomized clinical trials. Nine covered surgical or radiosurgical approaches and the remaining one a widely adopted prognostic score. Overall, 30 randomized clinical trials were published between 1990 and 2010, including those with phase II design and excluding duplicate publications, for example, after longer followup or with focus on secondary endpoints. Twenty of these randomized clinical trials were published before 2008. Their median number of citations was 110, range 13–1013, compared to 5-6 citations for all types of publications. Annual citation rate appeared to gradually increase during the first 2-3 years after publication before reaching high levels. *Conclusions*. A large variety of preclinical and clinical topics achieved high numbers of citations. However, areas such as quality of life, side effects, and end-of-life care were underrepresented. Efforts to increase their visibility might be warranted.

## 1. Introduction 

Development of brain metastases is a common problem in several subgroups of patients with malignant melanoma, lung, breast, and kidney cancer [1, 2]. Given the large number of patients with brain metastases and important consequences for individual patients and health care systems [3], intense research activity is directed towards prevention and treatment. Significant progress in clinical management has been made during the last two decades [4]. Both local and systemic treatment approaches have been gradually refined. Landmark phase III randomized trials provided the framework for these advances. Eventually, researchers attempt to publish their results in a way that ensures high visibility and allows for broad adoption of the progress achieved. Successful publication is desirable for several reasons related to investigators' career advancement, tenure track or likelihood of future funding, and might be defined by various measures. Impact factor of journals is a two-edged sword, for example, regarding its correlation with the true scientific or practical impact of let us say radiation technology or neurosurgery advances and the publication bias that strikes negative or inconclusive studies [5–9]. Article download rates might provide some indication for visibility and impact but will depend on presence and quantity of fees charged by the publisher. Another potential measure of quality and impact of research is the citation rate. Landmark or practice-changing research is likely to be cited by successor trials, editorials, review articles, meta-analyses, and guidelines. In our attempt to review the most significant publications relevant for the topics of treatment, diagnosis, and prevention of brain metastases, we relied on citation rates of articles published between 1990 and 2010. Information about highly cited article types can be useful for preparation of future research projects. Moreover, identification of underrepresented areas might facilitate efforts to increase their visibility. 

## 2. Methods

A systematic search of the abstract and citation database Scopus (Elsevier B.V., http://www.scopus.com/) by use of the key words “brain metastases,” “cerebral metastases,” “intracranial metastases,” “central nervous system metastases” or “secondary brain tumor” was performed on November 28th and 29th 2011. Publications related to metastases from extracranial solid tumors in pediatric and adult patients were selected irrespective of language and article type (case report, review, meta-analysis, etc.). In other words, all epidemiologic, diagnostic, therapeutic and preclinical topics were included. Prophylactic cranial irradiation and leptomeningeal carcinomatosis were not included unless for example, an article covered both leptomeningeal and parenchymal brain metastases. Articles dealing with brain metastases and glioma, for example, related to differential imaging diagnosis, were included as well. 

## 3. Results 

**Figure 1:**
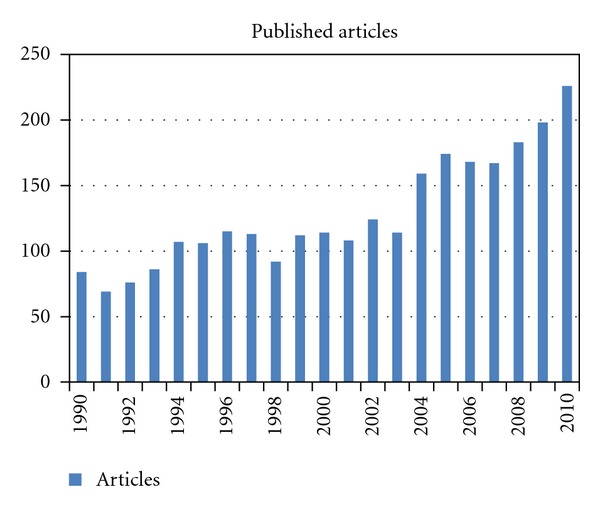
Number of articles published per year.

**Figure 2:**
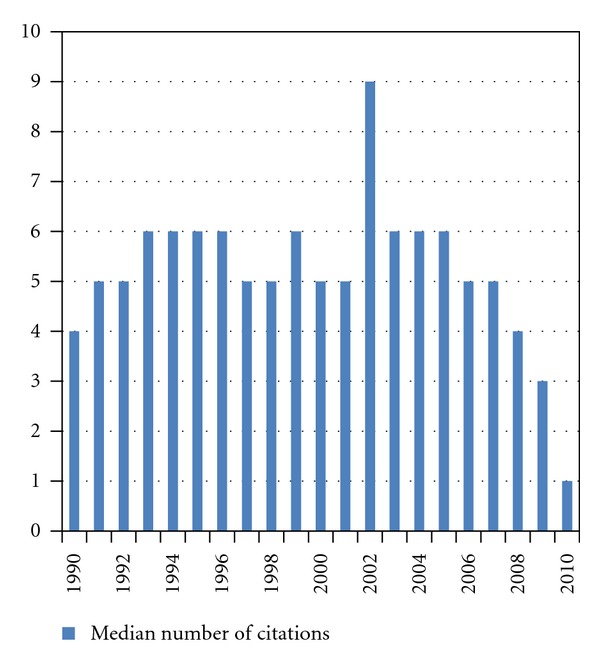
Median number of citations (basis: all articles published in a given year).

**Figure 3:**
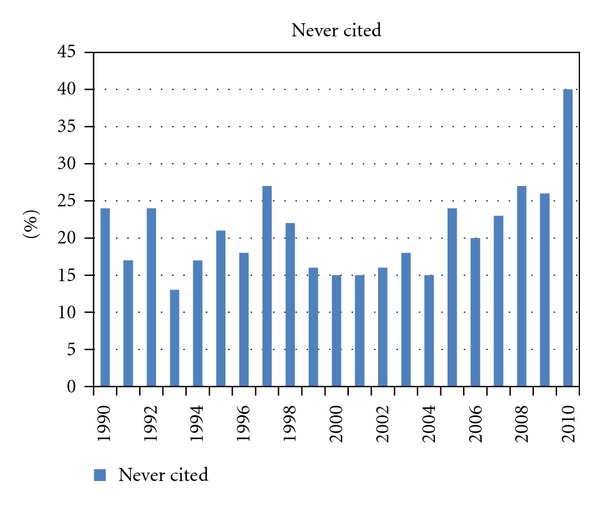
Percent of articles without any citation of all articles published in a given year.

**Figure 4:**
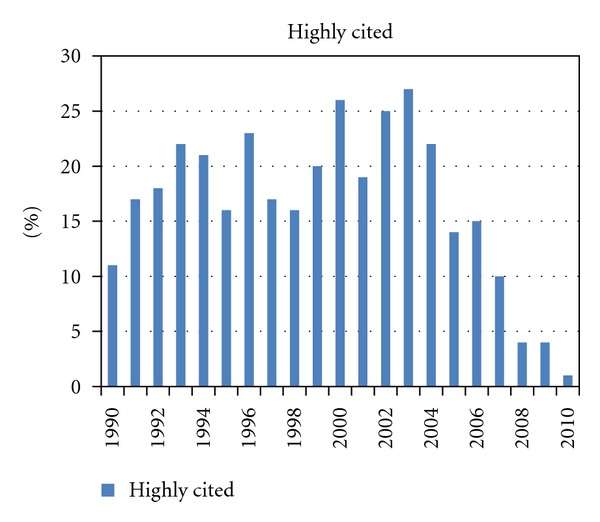
Percent of highly cited articles (>25 citations) of all articles published in a given year.

Overall 2695 publications were identified (69 to 226 per year).  Figure 1 shows the numbers of publications per year. After the year 2003, a consistent and substantial increase in the number of published articles is noted, underscoring a considerable increase in interest in this topic.  Figure 2 shows the median number of citations of all articles published in a given year (typically 5-6, lowest for recent years of publication). We also stratified all articles by number of citations (0, 1–5, 6–10, 11–25, 26–50, 51–100, >100). Except for the year 2002, most articles belonged to the group with 1–5 citations (24–35%, except for 42% in 2009 and 46% in 2010). In 2002, articles with 11–25 citations comprised the largest subgroup (24% of all articles).  Figure 3 shows the proportion of articles without any citation (typically between 15 and 25% of all articles published in a given year; 22% of all 2695 articles).  Figure 4 shows the proportion of highly cited articles, arbitrarily defined as more than 25 citations (typically between 15 and 25% of all articles published in a given year, except for recent years; 15% of all 2695 articles). 

**Figure 5:**
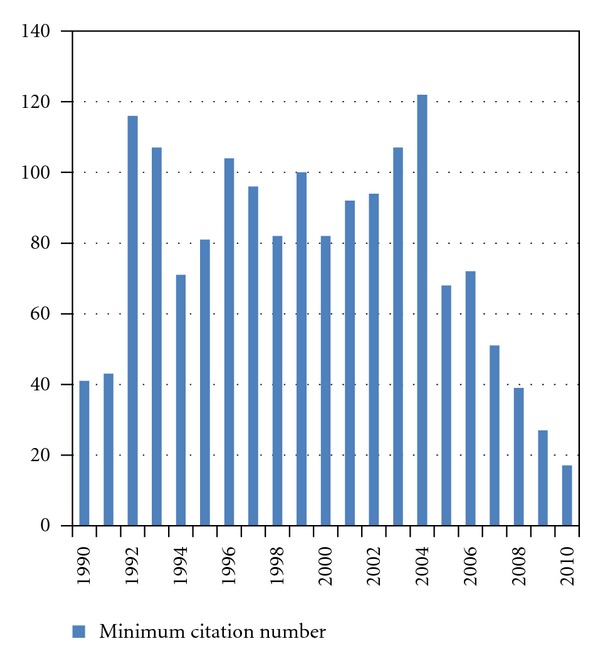
Minimum number of citations required to be among the 5 most cited articles in a given year.

References [10–116] represent the 5 most cited articles per year.  Figure 5 shows the minimum number of citations required to make it into the top 5 of each year (median 82, range 17–122).  Table 1 shows the 10 most cited articles overall. Seven of these report on randomized clinical trials. Nine covered surgical or radiosurgical approaches and the remaining one a widely adopted prognostic score. All were published before 2005. Since articles published, for example, in 1990 are more likely to have accumulated a large number of citations than articles published in 2010, the average of the annual numbers of citations was also calculated. For this purpose, 2011 was defined as 0.92 years (11 of 12 months; January–November).  Table 2 shows the 11 articles with most citations per year (several had 28 annual citations, which was the minimum number required for this endpoint). The table contains articles published between 1990 and 2009, but none of the 2010 publications had accumulated enough citations. Six of the articles reported on randomized clinical trials. The same number of publications covered surgical or radiosurgical approaches and 3 brain metastases in patients with breast cancer. 

Overall, 30 randomized clinical trials were published between 1990 and 2010, including those with phase II design and excluding duplicate publications, for example, after longer followup or with focus on secondary endpoints. Ten of these were published after 2008. Their median number of citations was 13.5, range 1–82. Twenty randomized clinical trials were published before 2008. Their median number of citations was 110, range 13–1013. The most cited articles (top five of each year [10–116]) were published in 35 different scientific journals. Twenty-five articles (23%) were published in the International Journal of Radiation Oncology, Biology, Physics, 14 (13%) in the Journal of Clinical Oncology, 12 (11%) in Cancer, 9 (8%) in the Journal of Neurosurgery, and 6 (6%) in the Journal of Neuro-Oncology.

## 4. Discussion

This overview is based on a systematic literature search where we decided to apply a broad definition of brain metastases-related publication. It should be kept in mind that not all completed research projects eventually will be published. We acknowledge that some of the selected articles might be subject to debate. In this overview, we focused on citation rate. Articles with high numbers of citations are likely those that impressed other clinicians/scientists and had profound influence on clinical practice or future developments in the field. However, the majority of published articles reviewed here received limited attention (22% were not cited at all). In a study covering the Lancet, JAMA, and New England Journal of Medicine, from October 1999 to March 2000, the authors found that presence of industry funding and an industry-favoring result was associated with an increase in annual citation rate of 25.7 (95% confidence interval, 8.5 to 42.8) compared to the absence of both industry funding and industry-favoring results [117]. Higher annual rates of citation were also associated with articles dealing with cardiovascular medicine (13.3 more; 95% confidence interval, 3.9 to 22.3) and oncology (12.6 more; 95% confidence interval, 1.2 to 24.0), articles with group authorship (11.1 more; 95% confidence interval, 2.7 to 19.5), larger sample size and journal of publication.

As stated in the previous section, we evaluated average annual citation rate because the exact time course or kinetics of citation is hard to predict and varies with topic and journal [118, 119]. Both accumulation of citations of recently published articles and reduced interest in older articles over time pose challenges if reliable quantitative analysis is attempted. We did not account for date of publication, that is, whether an article was published earlier or later during a given year. For the purpose of this overview, the chosen methods are sufficient. Of course, more detailed and quantitative analyses can be performed with the internet-based tools available. Self-citation is likely to influence the final citation count of some generally sparsely cited articles, whereas its impact on highly cited articles might be less pronounced. 

Our results are consistent with the assumption that citation rate is gradually increasing for approximately 2-3 years after publication. After several years with large numbers of citations, annual rates for older articles might decline. However, the purpose of this overview was not to explore dynamics of citation count. Given the fact that major scientific journals in the field, for example, Journal of Clinical Oncology and International Journal of Radiation Oncology, Biology, Physics, had steady increases in the number of published issues and articles over this time period and that each article contains a certain number of references, the increase in total numbers of publications over time is expected to lead to a parallel increase in citation rates. It is also interesting to note that highly cited research was published in a large number of different scientific journals with or without high impact factor, but always in the English language. 

 The large diversity of topics covering basically all clinical, preclinical, biological, and technical aspects of the field is noteworthy and mirrors the highly multidisciplinary approach towards brain metastases. Randomized trials, which often were performed by cooperative groups, in part nationwide or on an international level, were cited more often than other studies. This finding underlines the importance of continued support for this type of trials. There has also been major progress in uncovering patients at higher risk for development of brain metastases and predicting the outcome after treatment. In many instances, the latter was also achieved through multi-institutional cooperation. Research in areas such as quality of life, side effects, and end-of-life care was less likely to result in highly cited articles. While this does not mean that such research remains unrecognized or has a generally low likelihood of publication, efforts to increase the number of highly prestigious and cited articles might be warranted. 

## 5. Conclusions

Research activity has increased in the time period between 1990 and 2010, where a large number of highly cited and practice changing studies have been published. Randomized trials were overrepresented among highly cited studies. Multi-institutional and cooperative group projects contributed importantly to the advancement of the field and were likely to receive high citation counts. 

## Figures and Tables

**Table 1:** Articles with most citations (absolute count).

Authors and year of publication	Short title	Absolute citation count	Citations per year
Patchell et al. 1990 [10]	Randomized trial of surgery in the treatment of single metastases	1013	85
Gaspar et al. 1997 [11]	RTOG RPA	700	47
Andrews et al. 2004 [12]	Whole brain radiation therapy with or without stereotactic radiosurgery boost (RTOG 9508 randomised trial)	509	64
Patchell et al. 1998 [13]	Randomized trial of postoperative radiotherapy in the treatment of single metastases	487	35
Flickinger et al. 1994 [14]	Multi-institutional experience with stereotactic radiosurgery for solitary brain metastasis	398	22
Kondziolka et al. 1999 [15]	Stereotactic radiosurgery plus whole brain radiotherapy versus radiotherapy alone for patients with multiple brain metastases	396	31
Vecht et al. 1993 [16]	Radiotherapy alone or combined with neurosurgery in single metastases	382	20
Alexander III et al. 1995 [17]	Stereotactic radiosurgery for the definitive, noninvasive treatment of brain metastases	333	20
Noordijk et al. 1994 [18]	The choice of treatment of single brain metastasis should be based on extracranial tumor activity and age	328	18
Mintz et al. 1996 [19]	A randomized trial to assess surgery in addition to radiotherapy in patients with single cerebral metastasis	292	18

**Table 2:** Articles with most citations per year.

Authors and year of publication	Short title	Citations per year	Absolute citation count
Patchell et al. 1990 [10]	Randomized trial of surgery in the treatment of single metastases	85	1013
Andrews et al. 2004 [12]	Whole brain radiation therapy with or without stereotactic radiosurgery boost (RTOG 9508 randomised trial)	64	509
Aoyama et al. 2006 [20]	Randomized trial of stereotactic radiosurgery plus whole-brain radiation therapy versus stereotactic radiosurgery alone	48	282
Gaspar et al. 1997 [11]	RTOG RPA	47	700
Bos et al. 2009 [21]	Genes that mediate breast cancer metastasis to the brain	42	123
Patchell et al. 1998 [13]	Randomized trial of postoperative radiotherapy in the treatment of single metastases	35	487
Kondziolka et al. 1999 [15]	Stereotactic radiosurgery plus whole brain radiotherapy versus radiotherapy alone for patients with multiple brain metastases	31	396
Bendell et al. 2003 [22]	Central nervous system metastases in women who receive trastuzumab for metastatic breast carcinoma	31	276
Lin et al. 2009 [23]	Multicenter phase II study of lapatinib in patients with brain metastases from HER2-positive breast cancer	28	83
Chang et al. 2009 [24]	Neurocognition in patients with brain metastases treated with radiosurgery or radiosurgery plus whole-brain irradiation: a randomised trial	28	82
Lin et al. 2008 [25]	Phase II trial of lapatinib for brain metastases in patients with EGFR 2-positive breast cancer	28	108

## References

[1] Langer CJ, Mehta MP (2005). Current management of brain metastases, with a focus on systemic options. *Journal of Clinical Oncology*.

[2] Suh JH (2010). Stereotactic radiosurgery for the management of brain metastases. *The New England Journal of Medicine*.

[3] Nieder C, Norum J, Stemland JG, Dalhaug A (2010). Resource utilization in patients with brain metastases managed with best supportive care, radiotherapy and/or surgical resection: a markov analysis. *Oncology*.

[4] Nieder C, Spanne O, Mehta MP, Grosu AL, Geinitz H (2011). Presentation, patterns of care, and survival in patients with brain metastases: what has changed in the last 20 years?. *Cancer*.

[5] Kumar V, Upadhyay S, Medhi B (2009). Impact of the impact factor in biomedical research: its use and misuse. *Singapore Medical Journal*.

[6] Young NS, Ioannidis JPA, Al-Ubaydli O (2008). Why current publication practices may distort science. *PLoS Medicine*.

[7] Kanaan Z, Galandiuk S, Abby M (2011). The value of lesser-impact-factor surgical journals as a source of negative and inconclusive outcomes reporting. *Annals of Surgery*.

[8] Owlia P, Vasei M, Goliaei B, Nassiri I (2011). Normalized impact factor (NIF): an adjusted method for calculating the citation rate of biomedical journals. *Journal of Biomedical Informatics*.

[9] Valérie D, Gevenois PA (2010). Bibliometric idicators: quality masurements of sientific publication. *Radiology*.

[10] Patchell RA, Tibbs PA, Walsh JW (1990). A randomized trial of surgery in the treatment of single metastases to the brain. *The New England Journal of Medicine*.

[11] Gaspar L, Scott C, Rotman M (1997). Recursive partitioning analysis (RPA) of prognostic factors in three Radiation Therapy Oncology Group (RTOG) brain metastases trials. *International Journal of Radiation Oncology Biology Physics*.

[12] Andrews DW, Scott CB, Sperduto PW (2004). Whole brain radiation therapy with or without stereotactic radiosurgery boost for patients with one to three brain metastases: phase III results of the RTOG 9508 randomised trial. *The Lancet*.

[13] Patchell RA, Tibbs PA, Regine WF (1998). Postoperative radiotherapy in the treatment of single metastases to the brain: a randomized trial. *Journal of the American Medical Association*.

[14] Flickinger JC, Kondziolka D, Lunsford LD (1994). A multi-institutional experience with stereotactic radiosurgery for solitary brain metastasis. *International Journal of Radiation Oncology Biology Physics*.

[15] Kondziolka D, Patel A, Lunsford LD, Kassam A, Flickinger JC (1999). Stereotactic radiosurgery plus whole brain radiotherapy versus radiotherapy alone for patients with multiple brain metastases. *International Journal of Radiation Oncology Biology Physics*.

[16] Vecht CJ, Haaxma-Reiche H, Noordijk EM (1993). Treatment of single brain metastasis: radiotherapy alone or combined with neurosurgery?. *Annals of Neurology*.

[17] Alexander E, Moriarty TM, Davis RB (1995). Stereotactic radiosurgery for the definitive, noninvasive treatment of brain metastases. *Journal of the National Cancer Institute*.

[18] Noordijk EM, Vecht CJ, Haaxma-Reiche H (1994). The choice of treatment of single brain metastasis should be based on extracranial tumor activity and age. *International Journal of Radiation Oncology Biology Physics*.

[19] Mintz AH, Kestle J, Rathbone MP (1996). A randomized trial to assess the efficacy of surgery in addition to radiotherapy in patients with a single cerebral metastasis. *Cancer*.

[20] Aoyama H, Shirato H, Tago M (2006). Stereotactic radiosurgery plus whole-brain radiation therapy vs stereotactic radiosurgery alone for treatment of brain metastases: a randomized controlled trial. *Journal of the American Medical Association*.

[21] Bos PD, Zhang XHF, Nadal C (2009). Genes that mediate breast cancer metastasis to the brain. *Nature*.

[22] Bendell JC, Domchek SM, Burstein HJ (2003). Central nervous system metastases in women who receive trastuzumab-based therapy for metastatic breast carcinoma. *Cancer*.

[23] Lin NU, Diéras V, Paul D (2009). Multicenter phase II study of lapatinib in patients with brain metastases from HER2-positive breast cancer. *Clinical Cancer Research*.

[24] Chang EL, Wefel JS, Hess KR (2009). Neurocognition in patients with brain metastases treated with radiosurgery or radiosurgery plus whole-brain irradiation: a randomised controlled trial. *The Lancet Oncology*.

[25] Lin NU, Carey LA, Liu MC (2008). Phase II trial of lapatinib for brain metastases in patients with human epidermal growth factor receptor 2-positive breast cancer. *Journal of Clinical Oncology*.

[26] Jacquillat C, Khayat D, Banzet P (1990). Final report of the French multicenter phase II study of the nitrosourea fotemustine in 153 evaluable patients with disseminated malignant melanoma including patients with cerebral metastases. *Cancer*.

[27] Loeffler JS, Kooy HM, Wen PY (1990). The treatment of recurrent brain metastases with stereotactic radiosurgery. *Journal of Clinical Oncology*.

[28] Brega K, Robinson WA, Winston K, Wittenberg W (1990). Surgical treatment of brain metastases in malignant melanoma. *Cancer*.

[29] Twelves CJ, Souhami RL, Harper PG (1990). The response of cerebral metastases in small cell lung cancer to systemic chemotherapy. *British Journal of Cancer*.

[30] Zhang RD, Fidler IJ, Price JE (1991). Relative malignant potential of human breast carcinoma cell lines established from pleural effusions and a brain metastasis. *Invasion and Metastasis*.

[31] Davis PC, Hudgins PA, Peterman SB, Hoffman JC (1991). Diagnosis of cerebral metastases: double-dose delayed CT vs contrast-enhanced MR imaging. *American Journal of Neuroradiology*.

[32] Coffey RJ, Flickinger JC, Bissonette DJ, Lunsford LD (1991). Radiosurgery for solitary brain metastases using the cobalt-60 gamma unit: methods and results in 24 patients. *International Journal of Radiation Oncology Biology Physics*.

[33] Ushio Y, Arita N, Hayakawa T (1991). Chemotherapy of brain metastases from lung carcinoma: a controlled randomized study. *Neurosurgery*.

[34] Patchell RA (1991). Brain metastases. *Neurologic Clinics*.

[35] Mehta MP, Rozental JM, Levin AB (1992). Defining the role of radiosurgery in the management of brain metastases. *International Journal of Radiation Oncology Biology Physics*.

[36] Posner JB (1992). Management of brain metastases. *Revue Neurologique*.

[37] Adler JR, Cox RS, Kaplan I, Martin DP (1992). Stereotactic radiosurgical treatment of brain metastases. *Journal of Neurosurgery*.

[38] Fuller BG, Kaplan ID, Adler J, Cox RS, Bagshaw MA (1992). Stereotaxic radiosurgery for brain metastases: the importance of adjuvant whole brain irradiation. *International Journal of Radiation Oncology Biology Physics*.

[39] Boogerd W, Dalesio O, Bais EM, van der Sande JJ (1992). Response of brain metastases from breast cancer to systemic chemotherapy. *Cancer*.

[40] Bindal RK, Sawaya R, Leavens ME, Lee JJ (1993). Surgical treatment of multiple brain metastases. *Journal of Neurosurgery*.

[41] Engenhart R, Kimmig BN, Hover KH (1993). Long-term follow-up for brain metastases treated by percutaneous stereotactic single high-dose irradiation. *Cancer*.

[42] Somaza S, Kondziolka D, Lunsford LD, Kirkwood JM, Flickinger JC (1993). Stereotactic radiosurgery for cerebral metastatic melanoma. *Journal of Neurosurgery*.

[43] Kihlstrom L, Karlsson B, Lindquist C (1993). Gamma Knife surgery for cerebral metastases. Implications for survival based on 16 years experience. *Stereotactic and Functional Neurosurgery*.

[44] Vecht CJ, Hovestadt A, Verbiest HBC, van Vliet JJ, van Putten WLJ (1994). Dose-effect relationship of dexamethasone on Karnofsky performance in metastatic brain tumors: a randomized study of doses of 4, 8, and 16 mg per day. *Neurology*.

[45] Strugar J, Rothbart D, Harrington W, Criscuolo GR (1994). Vascular permeability factor in brain metastases: correlation with vasogenic brain edema and tumor angiogenesis. *Journal of Neurosurgery*.

[46] Schiff D, DeAngelis LM (1994). Therapy of venous thromboembolism in patients with brain metastases. *Cancer*.

[47] Wronski M, Arbit E, Burt M, Galicich JH (1995). Survival after surgical treatment of brain metastases from lung cancer: a follow-up study of 231 patients treated between 1976 and 1991. *Journal of Neurosurgery*.

[48] Rutigliano MJ, Lunsford LD, Kondziolka D (1995). The cost effectiveness of stereotactic radiosurgery versus surgical resection in the treatment of solitary metastatic brain tumors. *Neurosurgery*.

[49] Sijens PE, Knopp MV, Brunetti A (1995). 1H MR spectroscopy in patients with metastatic brain tumors: a multicenter study. *Magnetic Resonance in Medicine*.

[50] Phillips TL, Scott CB, Leibel SA, Rotman M, Weigensberg IJ (1995). Results of a randomized comparison of radiotherapy and bromodeoxyuridine with radiotherapy alone for brain metastases: report of RTOG trial 89-05. *International Journal of Radiation Oncology Biology Physics*.

[51] Yuh WTC, Tali ET, Nguyen HD, Simonson TM, Mayr NA, Fisher DJ (1995). The effect of contrast dose, imaging time, and lesion size in the MR detection of intracerebral metastasis. *American Journal of Neuroradiology*.

[52] Bindal RK, Sawaya R, Leavens ME, Hess KR, Taylor SH (1995). Reoperation for recurrent metastatic brain tumors. *Journal of Neurosurgery*.

[53] Nussbaum ES, Djalilian HR, Cho KH, Hall WA (1996). Brain metastases: histology, multiplicity, surgery, and survival. *Cancer*.

[54] Bindal AK, Bindal RK, Hess KR (1996). Surgery versus radiosurgery in the treatment of brain metastasis. *Journal of Neurosurgery*.

[55] Joseph J, Adler JR, Cox RS, Hancock SL (1996). Linear accelerator-based stereotaxic radiosurgery for brain metastases: the influence of number of lesions on survival. *Journal of Clinical Oncology*.

[56] Shaw E, Scott C, Souhami L (1996). Radiosurgery for the treatment of previously irradiated recurrent primary brain tumors and brain metastases: initial report of Radiation Therapy Oncology Group protocol 90-05. *International Journal of Radiation Oncology Biology Physics*.

[57] Shiau CY, Sneed PK, Shu HKG (1997). Radiosurgery for brain metastases: relationship of dose and pattern of enhancement to local control. *International Journal of Radiation Oncology Biology Physics*.

[58] Murray KJ, Scott C, Greenberg HM (1997). A randomized phase III study of accelerated hyperfractionation versus standard in patients with unresected brain metastases: a report of the Radiation Therapy Oncology Group (RTOG) 9104. *International Journal of Radiation Oncology Biology Physics*.

[59] Breneman JC, Warnick RE, Albright RE (1997). Stereotactic radiosurgery for the treatment of brain metastases: results of a single institution series. *Cancer*.

[60] Chiu AC, Delpassand ES, Sherman SI (1997). Prognosis and treatment of brain metastases in thyroid carcinoma. *Journal of Clinical Endocrinology and Metabolism*.

[61] Mori Y, Kondziolka D, Flickinger JC, Kirkwood JM, Agarwala S, Lunsford LD (1998). Stereotactic radiosurgery for cerebral metastatic melanoma: factors affecting local disease control and survival. *International Journal of Radiation Oncology Biology Physics*.

[62] Toda M, Rabkin SD, Martuza RL (1998). Treatment of human breast cancer in a brain metastatic model by G207, a replication-competent multimutated herpes simplex virus 1. *Human Gene Therapy*.

[63] Mori Y, Kondziolka D, Flickinger JC, Logan T, Lunsford LD (1998). Stereotactic radiosurgery for brain metastasis from renal cell carcinoma. *Cancer*.

[64] Agboola O, Benoit B, Cross P (1998). Prognostic factors derived from recursive partition analysis (RPA) of Radiation Therapy Oncology Group (RTOG) brain metastases trials applied to surgically resected and irradiated brain metastatic cases. *International Journal of Radiation Oncology Biology Physics*.

[65] Lagerwaard FJ, Levendag PC, Nowak PJCM, Eijkenboom WMH, Hanssens PEJ, Schmitz PIM (1999). Identification of prognostic factors in patients with brain metastases: a review of 1292 patients. *International Journal of Radiation Oncology Biology Physics*.

[66] Muacevic A, Kreth FW, Horstmann GA (1999). Surgery and radiotherapy compared with gamma knife radiosurgery in the treatment of solitary cerebral metastases of small diameter. *Journal of Neurosurgery*.

[67] Franciosi V, Cocconi G, Michiara M (1999). Front-line chemotherapy with cisplatin and etoposide for patients with brain metastases from breast carcinoma, nonsmall cell lung carcinoma, or malignant melanoma: a prospective study. *Cancer*.

[68] Lavine SD, Petrovich Z, Cohen-Gadol AA (1999). Gamma knife radiosurgery for metastatic melanoma: an analysis of survival, outcome, and complications. *Neurosurgery*.

[69] Shaw E, Scott C, Souhami L (2000). Single dose radiosurgical treatment of recurrent previously irradiated primary brain tumors and brain metastases: final report of RTOG protocol 90- 05. *International Journal of Radiation Oncology Biology Physics*.

[70] Gaspar LE, Scott C, Murray K, Curran W (2000). Validation of the RTOG recursive partitioning analysis (RPA) classification for brain metastases. *International Journal of Radiation Oncology Biology Physics*.

[71] Tsuda H, Takarabe T, Hasegawa F, Fukutomi T, Hirohashi S (2000). Large, central acellular zones indicating myoepithelial tumor differentiation in high-grade invasive ductal carcinomas as markers of predisposition to lung and brain metastases. *American Journal of Surgical Pathology*.

[72] Nieder C, Nestle U, Motaref B, Walter K, Niewald M, Schnabel K (2000). Prognostic factors in brain metastases: should patients be selected for aggressive treatment according to recursive partitioning analysis (RPA) classes?. *International Journal of Radiation Oncology Biology Physics*.

[73] Wroński M, Arbit E (2000). Surgical treatment of brain metastases from melanoma: a retrospective study of 91 patients. *Journal of Neurosurgery*.

[74] Sanghavi SN, Miranpuri SS, Chappell R (2001). Radiosurgery for patients with brain metastases: a multi-institutional analysis, stratified by the RTOG recursive partitioning analysis method. *International Journal of Radiation Oncology Biology Physics*.

[75] Abrey LE, Olson JD, Raizer JJ (2001). A phase II trial of temozolomide for patients with recurrent or progressive brain metastases. *Journal of Neuro-Oncology*.

[76] Christodoulou C, Bafaloukos D, Kosmidis P (2001). Phase II study of temozolomide in heavily pretreated cancer patients with brain metastases. *Annals of Oncology*.

[77] Carde P, Timmerman R, Mehta MP (2001). Multicenter phase Ib/II trial of the radiation enhancer motexafin gadolinium in patients with brain metastases. *Journal of Clinical Oncology*.

[78] Robinet G, Thomas P, Breton JL (2001). Results of a phase III study of early versus delayed whole brain radiotherapy with concurrent cisplatin and vinorelbine combination in inoperable brain metastasis of non-small-cell lung cancer: Groupe Français de Pneumo-Cancérologie (GFPC) protocol 95-1. *Annals of Oncology*.

[79] Antonadou D, Paraskevaidis M, Sarris G (2002). Phase II randomized trial of temozolomide and concurrent radiotherapy in patients with brain metastases. *Journal of Clinical Oncology*.

[80] Fidler IJ, Yano S, Zhang RD, Fujimaki T, Bucana CD (2002). The seed and soil hypothesis: vascularisation and brain metastases. *The Lancet Oncology*.

[81] Soffietti R, Ruda R, Mutani R (2002). Management of brain metastases. *Journal of Neurology*.

[82] Mehta MP, Shapiro WR, Glantz MJ (2002). Lead-in phase to randomized trial of motexafin gadolinium and whole-brain radiation for patients with brain metastases: centralized assessment of magnetic resonance imaging, neurocognitive, and neurologic end points. *Journal of Clinical Oncology*.

[83] Regine WF, Huhn JL, Patchell RA (2002). Risk of symptomatic brain tumor recurrence and neurologic deficit after radiosurgery alone in patients with newly diagonised brain metastases: results and implications. *International Journal of Radiation Oncology Biology Physics*.

[84] Mehta MP, Rodrigus P, Terhaard CHJ (2003). Survival and neurologic outcomes in a randomized trial of motexafin gadolinium and whole-brain radiation therapy in brain metastases. *Journal of Clinical Oncology*.

[85] Lu S, Ahn D, Johnson G, Cha S (2003). Peritumoral diffusion tensor imaging of high-grade gliomas and metastatic brain tumors. *American Journal of Neuroradiology*.

[86] Patchell RA (2003). The management of brain metastases. *Cancer Treatment Reviews*.

[87] O’Neill BP, Iturria NJ, Link MJ, Pollock BE, Ballman KV, O’Fallon JR (2003). A comparison of surgical resection and stereotactic radiosurgery in the treatment of solitary brain metastases. *International Journal of Radiation Oncology Biology Physics*.

[88] Clayton AJ, Danson S, Jolly S (2004). Incidence of cerebral metastases in patients treated with trastuzumab for metastatic breast cancer. *British Journal of Cancer*.

[89] Meyers CA, Smith JA, Bezjak A (2004). Neurocognitive function and progression in patients with brain metastases treated with whole-brain radiation and motexafin gadolinium: results of a randomized phase III trial. *Journal of Clinical Oncology*.

[90] Lin NU, Bellon JR, Winer EP (2004). CNS metastases in breast cancer. *Journal of Clinical Oncology*.

[91] Barnholtz-Sloan JS, Sloan AE, Davis FG, Vigneau FD, Lai P, Sawaya RE (2004). Incidence proportions of brain metastases in patients diagnosed (1973 to 2001) in the Metropolitan Detroit Cancer Surveillance System. *Journal of Clinical Oncology*.

[92] Mehta MP, Tsao MN, Whelan TJ (2005). The American Society for Therapeutic Radiology and Oncology (ASTRO) evidence-based review of the role of radiosurgery for brain metastases. *International Journal of Radiation Oncology Biology Physics*.

[93] Weil RJ, Palmieri DC, Bronder JL, Stark AM, Steeg PS (2005). Breast cancer metastasis to the central nervous system. *American Journal of Pathology*.

[94] Chiu CH, Tsai CM, Chen YM, Chiang SC, Liou JL, Perng RP (2005). Gefitinib is active in patients with brain metastases from non-small cell lung cancer and response is related to skin toxicity. *Lung Cancer*.

[95] Verger E, Gil M, Yaya R (2005). Temozolomide and concomitant whole brain radiotherapy in patients with brain metastases: a phase II randomized trial. *International Journal of Radiation Oncology Biology Physics*.

[96] Stark AM, Tongers K, Maass N, Mehdorn HM, Held-Feindt J (2005). Reduced metastasis-suppressor gene mRNA-expression in breast cancer brain metastases. *Journal of Cancer Research and Clinical Oncology*.

[97] Khuntia D, Brown P, Li J, Mehta MP (2006). Whole-brain radiotherapy in the management of brain metastasis. *Journal of Clinical Oncology*.

[98] Hicks DG, Short SM, Prescott NL (2006). Breast cancers with brain metastases are more likely to be estrogen receptor negative, express the basal cytokeratin CK5/6, and overexpress HER2 or EGFR. *American Journal of Surgical Pathology*.

[99] Tham YL, Sexton K, Kramer R, Hilsenbeck S, Elledge R (2006). Primary breast cancer phenotypes associated with propensity for central nervous system metastases. *Cancer*.

[100] Gabos Z, Sinha R, Hanson J (2006). Prognostic significance of human epidermal growth factor receptor positivity for the development of brain metastasis after newly diagnosed breast cancer. *Journal of Clinical Oncology*.

[101] Lin NU, Winer EP (2007). Brain metastases: the HER2 paradigm. *Clinical Cancer Research*.

[102] Aoyama H, Tago M, Kato N (2007). Neurocognitive function of patients with brain metastasis who received either whole brain radiotherapy plus stereotactic radiosurgery or radiosurgery alone. *International Journal of Radiation Oncology Biology Physics*.

[103] Stemmler HJ, Schmitt M, Willems A, Bernhard H, Harbeck N, Heinemann V (2007). Ratio of trastuzumab levels in serum and cerebrospinal fluid is altered in HER2-positive breast cancer patients with brain metastases and impairment of blood-brain barrier. *Anti-Cancer Drugs*.

[104] Li J, Bentzen SM, Renschler M, Mehta MP (2007). Regression after whole-brain radiation therapy for brain metastases correlates with survival and improved neurocognitive function. *Journal of Clinical Oncology*.

[105] Eichler AF, Loeffler JS (2007). Multidisciplinary management of brain metastases. *Oncologist*.

[106] Sperduto PW, Berkey B, Gaspar LE, Mehta M, Curran W (2008). A new prognostic index and comparison to three other indices for patients with brain metastases: an analysis of 1,960 patients in the RTOG database. *International Journal of Radiation Oncology Biology Physics*.

[107] Gril B, Palmieri D, Bronder JL (2008). Effect of lapatinib on the outgrowth of metastatic breast cancer cells to the brain. *Journal of the National Cancer Institute*.

[108] Muacevic A, Wowra B, Siefert A, Tonn JC, Steiger HJ, Kreth FW (2008). Microsurgery plus whole brain irradiation versus Gamma Knife surgery alone for treatment of single metastases to the brain: a randomized controlled multicentre phase III trial. *Journal of Neuro-Oncology*.

[109] Pouessel D, Culine S (2008). High frequency of intracerebral hemorrhage in metastatic renal carcinoma patients with brain metastases treated with tyrosine kinase inhibitors targeting the vascular endothelial growth factor receptor. *European Urology*.

[110] Socinski MA, Langer CJ, Huang JE (2009). Safety of bevacizumab in patients with non-small-cell lung cancer and brain metastases. *Journal of Clinical Oncology*.

[111] Mehta MP, Shapiro WR, Phan SC (2009). Motexafin gadolinium combined with prompt whole brain radiotherapy prolongs time to neurologic progression in non-small-cell lung cancer patients with brain metastases: results of a phase III trial. *International Journal of Radiation Oncology Biology Physics*.

[112] Kienast Y, von Baumgarten L, Fuhrmann M (2010). Real-time imaging reveals the single steps of brain metastasis formation. *Nature Medicine*.

[113] Linskey ME, Andrews DW, Asher AL (2010). The role of stereotactic radiosurgery in the management of patients with newly diagnosed brain metastases: a systematic review and evidence-based clinical practice guideline. *Journal of Neuro-Oncology*.

[114] Gaspar LE, Mehta MP, Patchell RA (2010). The role of whole brain radiation therapy in the management of newly diagnosed brain metastases: a systematic review and evidence-based clinical practice guideline. *Journal of Neuro-Oncology*.

[115] Kalkanis SN, Kondziolka D, Gaspar LE (2010). The role of surgical resection in the management of newly diagnosed brain metastases: a systematic review and evidence-based clinical practice guideline. *Journal of Neuro-Oncology*.

[116] Mehta MP, Paleologos NA, Mikkelsen T (2010). The role of chemotherapy in the management of newly diagnosed brain metastases: a systematic review and evidence-based clinical practice guideline. *Journal of Neuro-Oncology*.

[117] Figg WD, Dunn L, Liewehr DJ (2006). Scientific collaboration results in higher citation rates of published articles. *Pharmacotherapy*.

[118] Stringer MJ, Sales-Pardo M, Amaral LAN (2008). Effectiveness of journal ranking schemes as a tool for locating information. *PLoS ONE*.

[119] Stringer MJ, Sales-Pardo M, Nunes Amaral LA (2010). Statistical validation of a global model for the distribution of the ultimate number of citations accrued by papers published in a scientific journal. *Journal of the American Society for Information Science and Technology*.

